# STAT5 activation in B-cell acute lymphoblastic leukemia: damned if you do, damned if you don’t

**DOI:** 10.14800/ccm.1186

**Published:** 2016-02-22

**Authors:** Zhengqi Wang, Kevin D. Bunting

**Affiliations:** Department of Pediatrics, Aflac Cancer and Blood Disorders Center of Children’s Healthcare of Atlanta and Emory University School of Medicine, Atlanta, GA, United States

**Keywords:** cytokine signaling, JAK/STAT, hematopoiesis, lymphoid neoplasia, Myc, Bcl-2

## Abstract

A significant role of the microenvironment in leukemogenesis is beginning to emerge. The leukemia cell microenvironment consists of not only the stromal and endothelial cell components but also the normal hematopoietic cells. Signal transducer and activator of transcription 5 (STAT5) is a latent transcription factor that is normally transiently activated by phosphorylation in response to microenvironmental signals. In hematopoietic cells, persistently activated STAT5 via aberrant receptor signaling, Janus kinases (JAKs), or intracellular tyrosine kinases is a bona fide driver of leukemogenesis. However, active IL-7/STAT5 signaling also protects the early B-cell genome by suppressing error-prone recombination and vulnerability to transformation. Along these lines, we have reported that lymphocyte development from transplanted STAT5-deficient fetal liver cells was blocked at the pre-pro-B-cell stage but when combined with transgenic Myc and Bcl-2 promoted faster initiation of B-ALL. Furthermore, inflammatory responses may also be involved in leukemia initiation in both pediatric and adult patients which are associated with decreased phosphorylation of STAT5. Likewise, additional targeted agents continue to be developed for precision medicine that prominently suppress signaling pathways. A common theme of all of these perturbations is potential risk for dysregulating hematopoiesis through general transcriptional modulation. Here we discuss the potential for STAT5 inhibition as a double edged sword in certain hematologic disorders, such as early B-cell lymphoblastic leukemias. Considering the rapid pace of understanding of the pre-leukemic decrease in poly-clonality that precedes leukemia, the functional changes associated with microenvironmental influences are thus of potential clinical significance.

Cytokines that utilize the common gamma chain (γC) are critical for lymphoid development and function. Deficiency of interleukin (IL)-7, IL-7 receptor, γC, or Janus kinase 3 (JAK3) results in an early block in T and B lymphocyte development in mice ^[1]^ and transgenic Bcl-2 expression can restore T but not B lymphocyte development in this pathway ^[2–5]^. Activation of signal transducer and activator of transcription 5 (STAT5) by IL-7 plays an important role in murine lymphocyte development ^[6]^. Deletion of STAT5 blocks early development at the pre/pro-B cell stage and phenocopies that of the upstream defects, indicating that STAT5 is a major regulator of B-lineage development but not B-cell maturation or function ^[7]^.

Constitutive STAT5 activation is frequently observed in myeloid and lymphoid malignancies ^[8]^ and murine studies suggest that STAT5 is functionally important in certain types of B-cell acute lymphoblastic leukemia/lymphoma (B-ALL). The SL/Kh strain of mice develops spontaneous pre-B-ALL at more than 90% incidence by 6 months of age due to constitutive activation of STAT5a by retrovirus integration^[9]^ and transgenic overexpression of STAT5a^S711F^ cooperates with p53 deficiency to promote B-ALL ^[10]^. Loss of BLNK adapter protein causes autocrine JAK3/STAT5 activation and B-ALL in mice ^[11]^ and haploinsufficiency of EBF1 or PAX5 synergizes with activated STAT5 in ALL ^[12]^. Reciprocally, haploinsufficiency of STAT5 can attenuate IL-7 overexpression induced B-ALL by amelioration of IL-7 signal strength ^[13]^. Furthermore, STAT5 deficient B-cells were refractory to transformation via BCR-ABL ^[14, 15]^ and Tel-PDGFβ^[16]^ fusion proteins. Despite strong evidence for oncogenic activity in kinase-driven lymphoid leukemias, the role of STAT5 appears to be context dependent and subject to influences from the microenvironment in ways that are just beginning to be understood.

Interestingly, mass cytometry studies reveal interesting biology related to STAT5 activation in the B-cell lineage. Human IL-7 becomes uncoupled from STAT5 during pre/pro-B cell development and pSTAT5 becomes ligand independent at a time corresponding to VDJ recombination ^[17]^. A comparable result has been reported for murine IL-7 signaling using knockout mice ^[18]^. Decreased STAT5 activation also appears to be an important pre-leukemic change that predisposes the early B-cell genome to mutagenic complications due to increased expression of AID and RAG genes ^[19]^. Reduced IL-7/STAT5 signal strength is associated with development of B-ALL in children ^[19]^ and adults ^[20]^ respectively. Inflammation during the neonatal period or during aging can be associated with STAT5 suppression and leukemic transformation ^[19, 21]^, although potentially due to entirely different mechanisms. Because of the strong correlation between decreased STAT5 activity and B-cell leukemia, we were interested in exploring the functional relevance of STAT5 using a knockout mouse strategy. This approach was done using complete germline knockout as well as interferon-inducible conditional knockout of STAT5 in adult mice.

To study the role of STAT5 in lymphoid development, we utilized a classical model of Myc/Bcl-2 initiated murine B-cell leukemia ^[22]^ using congenic mouse strains (CD45.1 and CD45.2). We isolated CD45.2 positive, E14.5 wild-type, STAT5ab^null/null^, H2K-Bcl-2 ^[23]^, or H2K-Bcl-2/STAT5ab^null/null^ fetal liver (FL) cells from STAT5ab^+/null^ x H2K-Bcl-2/STAT5ab^+/null^ crosses. FL cells were transplanted into lethally-irradiated CD45.1 positive Boy J recipients. Recipient mice were bled 16 weeks after transplantation and absolute hematology counts were determined. The number of donor-derived Gr-1^+^ cells was very similar among all four groups of transplanted mice. However, the absolute number of donor-derived B220^+^ and CD4^+^ cells in STAT5ab^null/null^ mice was significantly reduced to 2.2% and 2.5% respectively compared to the wild type transplanted mice. In contrast, the absolute number of donor derived B220^+^ and CD4^+^ cells in H2K-Bcl-2 FL transplanted mice was elevated 2.8- and 2.5-fold respectively compared to the wild type transplanted mice. Strikingly, in the absence of STAT5ab, H2K-Bcl-2 FL-transplanted mice had little donor contribution to B220^+^ and CD4^+^ cells. Furthermore, we also transplanted the same set of FL cells into lethally irradiated common γC^−/−^ mice which lack T, B and NK cells and identical results were obtained. These data suggest that STAT5-dependent lymphocyte development cannot be corrected by the expression of H2K-Bcl-2. It is worthy to note that STAT5 deficiency does not block B cell development completely. There was a residual amount of B220^+^ cells in the spleen (weight was 20% of wild-type) of mice receiving transplantation with STAT5ab^null/null^ FL cells. Likewise, we recently reported using STAT5ab^ΔN/ΔN^ mice that hypomorphic expression of N-terminal truncated STAT5 and the accompanying leakiness in B-lineage development, permits substantial rescue of B-cell numbers with overexpression of Bcl-2 alone ^[24]^.

Deregulated c-Myc is a naturally occurring secondary event known to complement Bcl-2 in mouse and human lymphomagenesis. Eμ-Myc and Bcl-2 have a synergistic effect in lymphomagenesis. Since STAT5 deficiency severely impairs B cell development even in the presence of expression of H2K-Bcl-2, we set out to test the role of STAT5 in Eμ-Myc initiated B-ALL ^[5]^. We isolated CD45.2 positive, E14.5 Eμ-Myc, H2K-Bcl-2, and Eμ-Myc/H2K-Bcl-2 FL cells with or without STAT5 from Eμ-Myc/STAT5ab^+/null^ x H2K-Bcl-2/STAT5ab^+/null^ crosses. FL cells were transplanted into lethally irradiated Boy J recipients (CD45.1). Recipient mice were bled at 4 weeks after the transplantation and peripheral blood hematology was analyzed. In the presence of STAT5, Eμ-Myc FL-transplanted mice had a relatively normal number of B220^+^ cells whereas H2K-Bcl-2 transplanted mice had increased B220^+^ cells. Eμ-Myc/H2K-Bcl-2 transplanted mice developed B-ALL. In the absence of STAT5, neither Eμ-Myc nor H2K-Bcl-2 FL-transplanted mice had more B220^+^ cells than that of STAT5ab^null/null^ FL-transplanted mice. Eμ-Myc/H2K-Bcl-2/STAT5ab^null/null^ FL developed B-ALL rapidly resulting in elevated B cell counts that were even higher than that of wild-type mice. As expected, expanded B cells were blocked at pre/pro-B cell stages. Eμ-Myc/H2K-Bcl-2/STAT5ab^null/null^ FL-transplanted mice also had a median survival of 44 days compared to 91 days in the presence of STAT5 (P<0.001).

Since Eμ-Myc FL-transplanted mice not expressing H2K-Bcl-2 developed B-ALL with a long median onset of 49 weeks, we also tested Myc-initiated B-ALL using conditional deletion of STAT5 in adult mice. Interferon-induced conditional deletion of STAT5 alone did not present toxicity and resulted in a high level of deletion of the floxed STAT5ab locus in circulating Gr-1^+^ leukocytes and significantly decreased circulating B220^+^ cells as described, without affecting mouse survival. Consistent with the FL transplant model, conditional deletion of STAT5 in the adult Eμ-Myc mice led to a shorter latency (112 days) than that of control mice (> 223 days; P=0.05).

Overall, these data demonstrate that although STAT5 activation underlies normal IL-7 driven B-cell expansion, loss of STAT5 cannot block Eμ-Myc induced lymphomagenesis but instead accelerates the initiation of B-ALL (Fig. 1). This result is similar to phospholipase Cγ2 deficiency which also impedes early B-cell development but can accelerate Myc-mediated lymphomagenesis ^[25]^. We found that STAT5 regulates lymphoid commitment genes and HSC quiescence/self-renewal genes in a reciprocal manner ^[5]^. The reciprocal relationship between these gene expression programs has been proposed as a potential mechanism to be exploited for human HSC expansion ^[26]^. STAT5 inhibition with small molecules has become a major focus in the field and the number of drugs with the ability to suppress STAT5 has been growing. Because transcription factors regulate many genes, some positively and some negatively, the impact of many signaling inhibitors on downstream gene expression can be unpredictable. Since most drugs are active on both normal and leukemic cells, the “on-target” effects of highly sensitive drugs also needs to be considered.

The microenvironment of the leukemia not only consists of the stromal elements and endothelial cells, but also the normal hematopoietic cells. Our study and the work of others suggest the possibility of dysregulated hematopoiesis and clonal evolution during leukemogenesis in the setting of perturbation such as aging, hematologic stress in the form of irradiation, cytotoxic drugs, and possibly signaling inhibitors (Fig. 2). Relapsed leukemia is a major problem for both pediatric and adult patients who initially respond to treatment.

Growing evidence suggests that hematopoiesis is driven by many clones that turn on and off to sustain normal blood cell development ^[27]^. Skewing this normal distribution of blood lineages may have unintended consequences and could conceivably activate dormant clones or establish new waves of clonality with unfavorable potential. Such activation has been described following chemotherapy treatment in AML patients ^[28, 29]^. Clearly more work needs to be done in this area with combined clonal and functional analyses using both mouse models and human leukemia systems. Clonal heterogeneity of indeterminate significance ^[30, 31]^ has been described and although it doesn’t warrant immediate changes to patient care, it is being monitored carefully in a variety of settings. Along these lines, genotypes of patients to be treated with targeted agents may also need to be evaluated carefully to reduce chances that treatment imposes additional clonal variation. Characterization of inflammatory stromal cells and inflammatory mediators, endothelial cell niches and “normal” hematopoiesis would also be required to fully understand the impact of the diverse microenvironment on leukemia evolution.

## Figures and Tables

**Figure 1:**
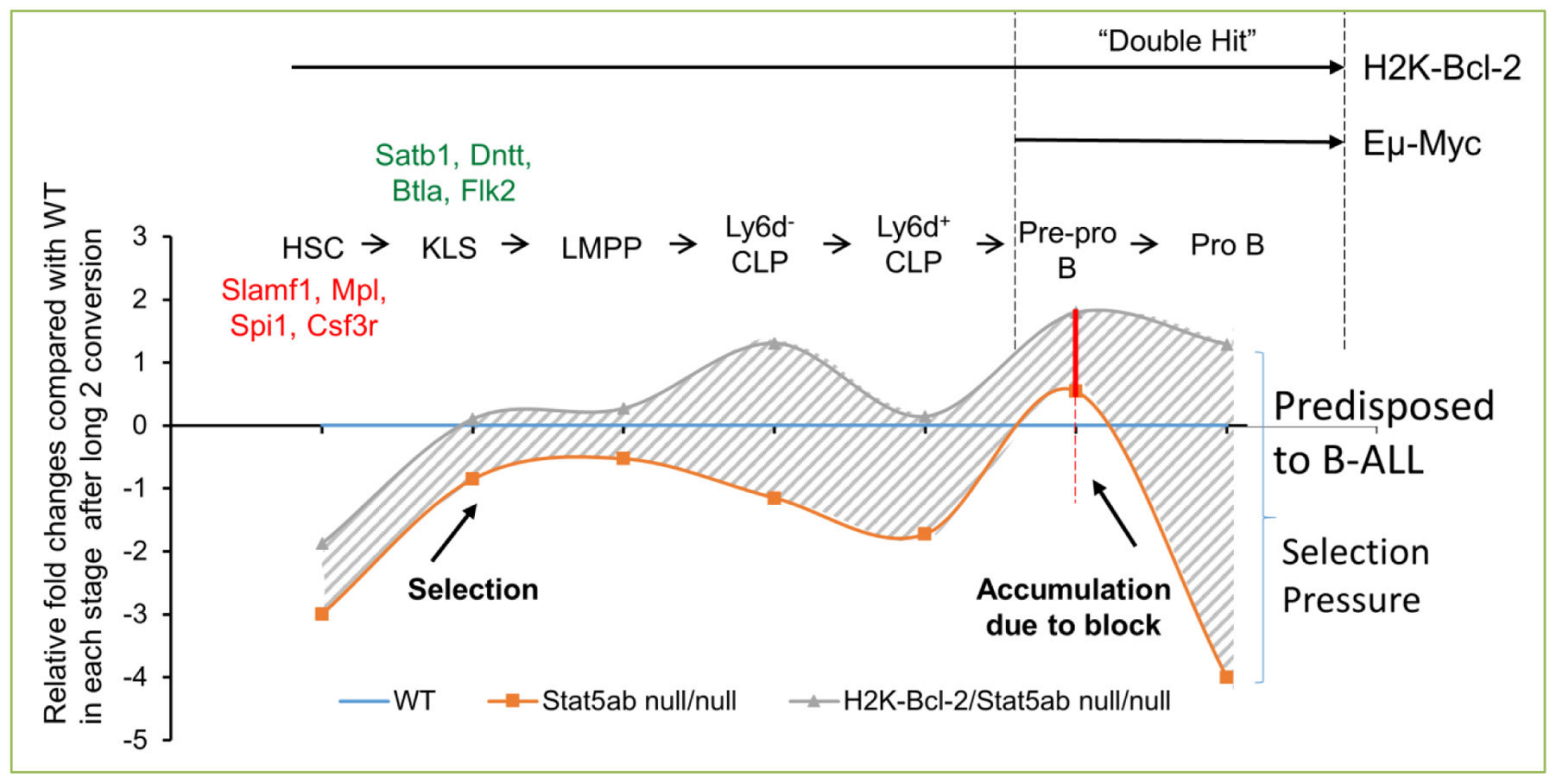
Pre-leukemic selection pressure in hematopoiesis induced by deletion of STAT5 alone or in combination with Myc/Bcl-2 The development of early B-cell stages is listed on the top. Relative fold changes for wild-type (blue line), STAT5ab^null/null^ (orange line), and STAT5ab^null/null^ + Bcl-2 transgene (gray line) were analyzed for B-cell development and leukemogenesis. The curves are a graphical representation of the data previously reported^[5]^. Data were normalized to the wild-type absolute number of cells at each stage of differentiation. STAT5 was found to play a major role in maintenance of the long-term repopulating hematopoietic stem cell (LT-HSC) pool but permits development toward the lymphoid-primed multipotent progenitor (LMPP). However, B-cell precursor development is sharply blocked at the Pre-pro-B-cell stage. Expression of Bcl-2 at each stage of development increases the absolute number of cells resulting in dramatic increases at the Pre-pro to Pro-B stages which also express Eμ-Myc. Overall, increased flux from early stem/progenitors results in more potential B-cell precursors that are susceptible to leukemic transformation.

**Figure 2:**
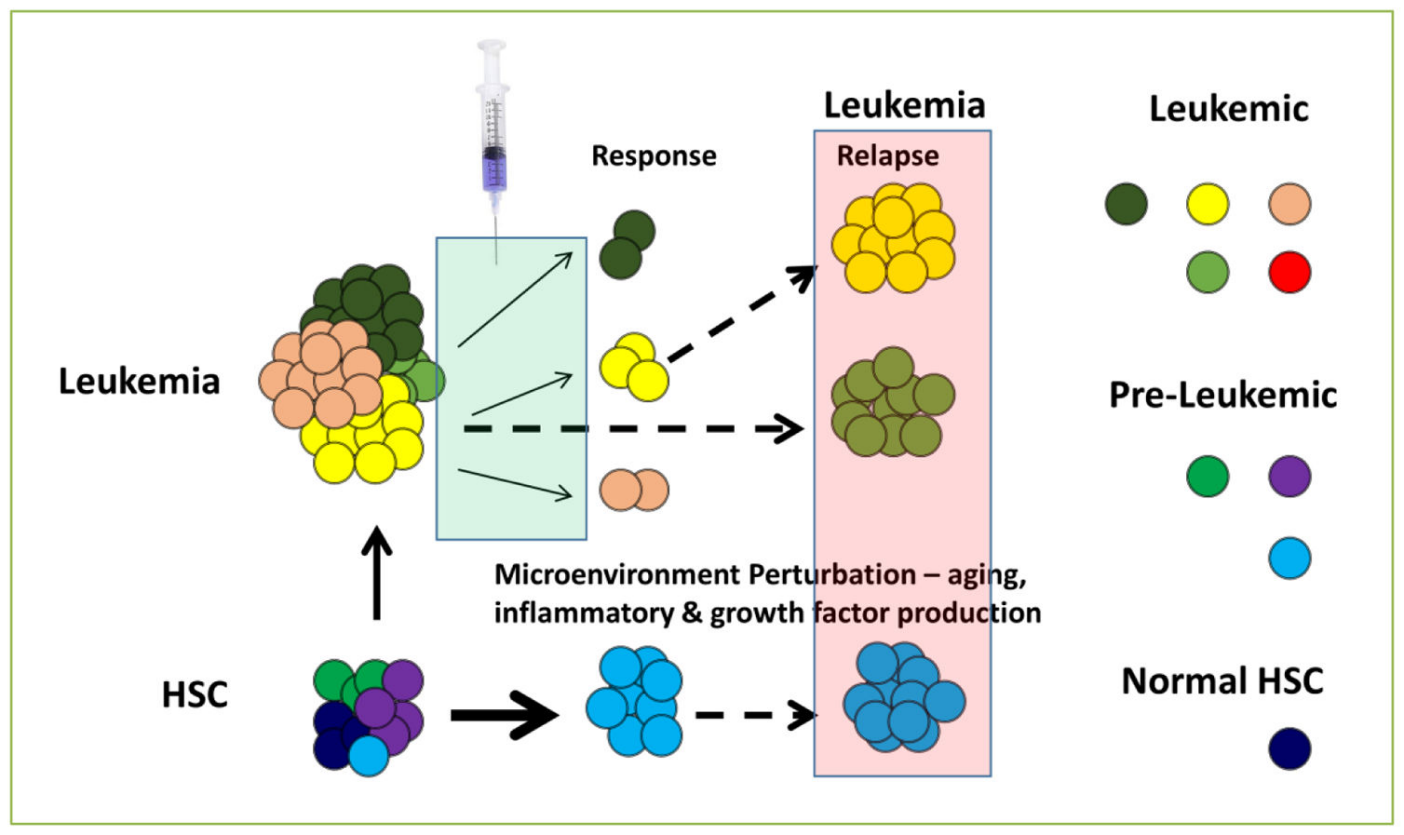
Potential routes from hematopoietic stem cell to leukemic stem cell Normal hematopoietic stem cells (HSC) acquire mutations with age due to environmental exposure and error-prone DNA replication/repair. HSCs can also be exposed to a variety of microenvironmental perturbations that predominate with age, including increased inflammatory signaling and cytokine/chemokine production from stromal, endothelial, or hematopoietic origin. The combination of these factors has the potential to promote leukemogenesis by facilitating transformation of HSC clones with pre-existing mutations. Likewise, already established leukemia can be treated with a growing number of targeted agents as part of a precision medicine strategy. However, this has the still unexplored potential of altering clonal dynamics leading to emergence of new clones with strong potential for relapse. These possibilities are now feasible to test using existing methodologies and it will be important to fully assess the side effects of new therapies as well as explore approaches to mitigate the microenvironmental changes that may co-exist and impact upon clonal evolution.

## References

[1] Uribe L, Weinberg KI (1998). X-linked SCID and other defects of cytokine pathways. SeminHematol.

[2] Maraskovsky E, O’Reilly LA, Teepe M, Corcoran LM, Peschon JJ, Strasser A (1997). Bcl-2 can rescue T lymphocyte development in interleukin-7 receptor-deficient mice but not in mutant rag-1−/− mice. Cell.

[3] Akashi K, Kondo M, von Freeden-Jeffry U, Murray R, Weissman IL (1997). Bcl-2 rescues T lymphopoiesis in interleukin-7 receptor-deficient mice. Cell.

[4] Kondo M, Akashi K, Domen J, Sugamura K, Weissman IL (1997). Bcl-2 rescues T lymphopoiesis, but not B or NK cell development, in common gamma chain-deficient mice. Immunity.

[5] Wang Z, Medrzycki M, Bunting ST, Bunting KD (2015). Stat5-deficient hematopoiesis is permissive for Myc-induced B-cell leukemogenesis. Oncotarget.

[6] Yao Z, Cui Y, Watford WT, Bream JH, Yamaoka K, Hissong BD (2006). Stat5a/b are essential for normal lymphoid development and differentiation. Proc Natl Acad Sci USA.

[7] Dai X, Chen Y, Di L, Podd A, Li G, Bunting KD (2007). Stat5 is essential for early B cell development but not for B cell maturation and function. JImmunol.

[8] Benekli M, Baer MR, Baumann H, Wetzler M (2003). Signal transducer and activator of transcription proteins in leukemias. Blood.

[9] Tsuruyama T, Nakamura T, Jin G, Ozeki M, Yamada Y, Hiai H (2002). Constitutive activation of Stat5a by retrovirus integration in early pre-B lymphomas of SL/Kh strain mice. Proc Natl Acad Sci USA.

[10] Joliot V, Cormier F, Medyouf H, Alcalde H, Ghysdael J (2006). Constitutive STAT5 activation specifically cooperates with the loss of p53 function in B-cell lymphomagenesis. Oncogene.

[11] Nakayama J, Yamamoto M, Hayashi K, Satoh H, Bundo K, Kubo M (2009). BLNK suppresses pre-B-cell leukemogenesis through inhibition of JAK3. Blood.

[12] Heltemes-Harris LM, Willette MJ, Ramsey LB, Qiu YH, Neeley ES, Zhang N (2011). Ebf1 or Pax5 haploinsufficiency synergizes with STAT5 activation to initiate acute lymphoblastic leukemia. J Exp Med.

[13] Abraham N, Ma MC, Snow JW, Miners MJ, Herndier BG, Goldsmith MA (2005). Haploinsufficiency identifies STAT5 as a modifier of IL-7-induced lymphomas. Oncogene.

[14] Hoelbl A, Schuster C, Kovacic B, Zhu B, Wickre M, Hoelzl MA (2010). Stat5 is indispensable for the maintenance of bcr/abl-positive leukaemia. EMBO MolMed.

[15] Hoelbl A, Kovacic B, Kerenyi MA, Simma O, Warsch W, Cui Y (2006). Clarifying the role of Stat5 in lymphoid development and Abelson induced transformation. Blood.

[16] Cain JA, Xiang Z, O’Neal J, Kreisel F, Colson A, Luo H (2007). Myeloproliferative disease induced by TEL-PDGFRB displays dynamic range sensitivity to Stat5 gene dosage. Blood.

[17] Bendall SC, Davis KL, Amir e, Tadmor MD, Simonds EF, Chen TJ (2014). Single-cell trajectory detection uncovers progression and regulatory coordination in human B cell development. Cell.

[18] Malin S, McManus S, Cobaleda C, Novatchkova M, Delogu A, Bouillet P (2010). Role of STAT5 in controlling cell survival and immunoglobulin gene recombination during pro-B cell development. NatImmunol.

[19] Swaminathan S, Klemm L, Park E, Papaemmanuil E, Ford A, Kweon SM (2015). Mechanisms of clonal evolution in childhood acute lymphoblastic leukemia. Nat Immunol.

[20] Henry CJ, Marusyk A, Zaberezhnyy V, Adane B, Degregori J (2010). Declining lymphoid progenitor fitness promotes aging-associated leukemogenesis. Proc Natl Acad Sci USA.

[21] Henry CJ, Casas-Selves M, Kim J, Zaberezhnyy V, Aghili L, Daniel AE (2015). Aging-associated inflammation promotes selection for adaptive oncogenic events in B cell progenitors. J Clin Invest.

[22] Strasser A, Harris AW, Bath ML, Cory S (1990). Novel primitive lymphoid tumours induced in transgenic mice by cooperation between myc and bcl-2. Nature.

[23] Domen J, Weissman IL (2000). Hematopoietic stem cells need two signals to prevent apoptosis; BCL-2 can provide one of these, Kitl/c-Kit signaling the other. J Exp Med.

[24] Wang Z, Li G, Bunting KD (2014). STAT5 N-domain deleted isoforms are naturally occurring hypomorphs partially rescued in hematopoiesis by transgenic Bcl-2 expression. Am J Blood Res.

[25] Wen R, Chen Y, Bai L, Fu G, Schuman J, Dai X (2006). Essential role of phospholipase C gamma 2 in early B-cell development and Myc-mediated lymphomagenesis. MolCell Biol.

[26] van GP, Kreso A, Wienholds E, Laurenti E, Eppert K, Lechman ER (2014). Reduced lymphoid lineage priming promotes human hematopoietic stem cell expansion. Cell Stem Cell.

[27] Sun J, Ramos A, Chapman B, Johnnidis JB, Le L, Ho YJ (2014). Clonal dynamics of native haematopoiesis. Nature.

[28] Walter MJ, Shen D, Ding L, Shao J, Koboldt DC, Chen K (2012). Clonal architecture of secondary acute myeloid leukemia. NEnglJ Med.

[29] Shlush LI, Zandi S, Mitchell A, Chen WC, Brandwein JM, Gupta V (2014). Identification of pre-leukaemic haematopoietic stem cells in acute leukaemia. Nature.

[30] Steensma DP, Bejar R, Jaiswal S, Lindsley RC, Sekeres MA, Hasserjian RP (2015). Clonal hematopoiesis of indeterminate potential and its distinction from myelodysplastic syndromes. Blood.

[31] Jaiswal S, Fontanillas P, Flannick J, Manning A, Grauman PV, Mar BG (2014). Age-related clonal hematopoiesis associated with adverse outcomes. N Engl J Med.

